# *A priori* prediction of response in multicentre locally advanced breast cancer (LABC) patients using quantitative ultrasound and derivative texture methods

**DOI:** 10.18632/oncotarget.27867

**Published:** 2021-01-19

**Authors:** Laurentius O. Osapoetra, Lakshmanan Sannachi, Karina Quiaoit, Archya Dasgupta, Daniel DiCenzo, Kashuf Fatima, Frances Wright, Robert Dinniwell, Maureen Trudeau, Sonal Gandhi, William Tran, Michael C. Kolios, Wei Yang, Gregory J. Czarnota

**Affiliations:** ^1^Department of Radiation Oncology, Sunnybrook Health Sciences Centre, Toronto, ON, Canada; ^2^Department of Radiation Oncology, University of Toronto, Toronto, ON, Canada; ^3^Physical Sciences, Sunnybrook Research Institute, Toronto, ON, Canada; ^4^Department of Medical Biophysics, University of Toronto, Toronto, ON, Canada; ^5^Department of Surgical Oncology, Department of Surgery, Sunnybrook Health Sciences Centre, Toronto, ON, Canada; ^6^Department of Surgery, University of Toronto, Toronto, ON, Canada; ^7^Department of Radiation Oncology, Princess Margaret Hospital, University Health Network, Toronto, ON, Canada; ^8^Radiation Oncology, London Health Sciences Centre, London, ON, Canada; ^9^Department of Oncology, Schulich School of Medicine and Dentistry, Western University, London, ON, Canada; ^10^Medical Oncology, Department of Medicine, Sunnybrook Health Sciences Centre, Toronto, ON, Canada; ^11^Department of Medicine, University of Toronto, Toronto, ON, Canada; ^12^Evaluative Clinical Sciences, Sunnybrook Research Institute, Toronto, ON, Canada; ^13^Department of Physics, Ryerson University, Toronto, ON, Canada; ^14^Department of Diagnostic Radiology, University of Texas, Houston, Texas, USA

**Keywords:** radiomics, breast cancer, texture-derivate, quantitative ultrasound, neoadjuvant chemotherapy

## Abstract

Purpose: We develop a multi-centric response predictive model using QUS spectral parametric imaging and novel texture-derivate methods for determining tumour responses to neoadjuvant chemotherapy (NAC) prior to therapy initiation.

Materials and Methods: QUS Spectroscopy provided parametric images of mid-band-fit (MBF), spectral-slope (SS), spectral-intercept (SI), average-scatterer-diameter (ASD), and average-acoustic-concentration (AAC) in 78 patients with locally advanced breast cancer (LABC) undergoing NAC. Ultrasound radiofrequency data were collected from Sunnybrook Health Sciences Center (SHSC), University of Texas MD Anderson Cancer Center (MD-ACC), and St. Michaels Hospital (SMH) using two different systems. Texture analysis was used to quantify heterogeneities of QUS parametric images. Further, a second-pass texture analysis was applied to obtain texture-derivate features. QUS, texture- and texture-derivate parameters were determined from both tumour core and a 5-mm tumour margin and were used in comparison to histopathological analysis for developing a response predictive model to classify responders versus non-responders. Model performance was assessed using leave-one-out cross-validation. Three standard classification algorithms including a linear discriminant analysis (LDA), k-nearest-neighbors (KNN), and support vector machines-radial basis function (SVM-RBF) were evaluated.

Results: A combination of tumour core and margin classification resulted in a peak response prediction performance of 88% sensitivity, 78% specificity, 84% accuracy, 0.86 AUC, 84% PPV, and 83% NPV, achieved using the SVM-RBF classification algorithm. Other parameters and classifiers performed less well running from 66% to 80% accuracy.

Conclusions: A QUS-based framework and novel texture-derivative method enabled accurate prediction of responses to NAC. Multi-centric response predictive model provides indications of the robustness of the approach to variations due to different ultrasound systems and acquisition parameters.

## INTRODUCTION

Locally advanced breast cancer (LABC) is an aggressive type of breast cancer with a wide range of clinical presentations [1, 2]. Any tumour that is greater than 5 cm or that involves the skin and the chest wall is locally advanced [1, 2]. Locally advanced breast cancer also includes inflammatory breast cancer and patients with fixed axillary lymph nodes or ipsilateral supraclavicular, infraclavicular, or internal mammary nodal involvement [1, 2]. Locally advanced breast cancer tumours remain a challenging clinical problem as most patients with locally advanced diseases have poorer long-term survival compared to those with early stage breast diseases [1, 2]. The standard treatment for LABC includes a multimodality treatment comprised of systemic therapy, surgery, and radiotherapy [1–3]. Neoadjuvant chemotherapy (NAC) facilitates tumour shrinkage, thus allowing inoperable tumours to be resected in selected patients. This is followed by surgery and adjuvant radiotherapy and targeted therapy or endocrinal therapy as indicated [4]. There continue to be variable tumour responses in patients with LABC receiving NAC, with only 15–40% ultimately achieving pathological complete response to therapy [3]. Several studies have demonstrated that tumour pathological response to NAC is an important prognostic factor for long-term disease-free survival (DFS) and overall survival (OS) in specific group of patients [5, 6]. Treatment response of LABC to NAC is conventionally evaluated at the conclusion of treatment, several months after treatment initiation. This evaluation is based on pathology assessments commonly using a Miller-Payne (MP) grading system that assesses tumour cellularity between pre-treatment core needle biopsies and post-treatment surgical specimens [6, 7]. Imaging biomarkers that can predict tumour responses at early stages NAC could guide individualized treatments.

Different characteristics between responsive and non-responsive tumors have been elucidated using histopathological analysis and radiomics. Histopathology assesses tumor cells proliferation and hormone receptor status. On the other hand, radiomics utilizes advanced computer-based image analysis and machine learning techniques to extract non-invasive imaging biomarkers and interpret data [8–10]. As an emerging field in medicine and oncology, radiomics allows for the inference of biological characteristics associated with treatment [8–10]. Tumours that are responsive to chemotherapy were shown to present less cell proliferation compared to those of non-responsive tumours, as the result of increased apoptosis [11, 12]. Human epidermal growth factor receptor 2 (HER2) expression has been shown to correlate with response to NAC, with HER2-positive tumours demonstrating significantly higher rates of achieving pathological complete response than those of HER2-normal tumours [13]. A study that used diffuse optical spectroscopic tomography (DOST) techniques for LABC patients identified significantly higher haemoglobin contents in patients with complete pathological response compared to those with incomplete pathological response [14]. Other studies that utilized magnetic resonance imaging (MRI), DOST [15], and circulating DNA and RNA-integrity measurements [16] only predicted responses after the initiation of chemotherapy.

Quantitative ultrasound (QUS) spectroscopy has been used for characterizing different types of tissue [17–23], classifying tissue abnormalities, differentiating benign from malignant tumours [24–26], and assessing tumour responses to treatment [27–32]. In pre-clinical studies, QUS spectroscopy has been used to differentiate benign versus malignant breast tumours and to differentiate types of mammary cancers [33, 34]. QUS spectroscopy performs spectral analysis of the raw radiofrequency (RF) signal used in generating ultrasound images and determines acoustic scattering parameters that reflect tissue microstructures. This technique compensates for ultrasound attenuation due to propagation through intervening tissue layers and the tumor. In addition, a normalization procedure was performed such that the measured frequency-dependent attenuation-corrected normalized power spectrum (NPS) or back-scattering coefficient (BSC) truly reflects the characteristics of tissue microstructures [35–37]. These microstructures are different between tumours that are responsive and non-responsive to NAC as supported by histopathological analysis [5–7]. Linear fit to the attenuation-corrected NPS allowed estimation of mid-band fit (MBF), spectral slope (SS), and 0-MHz spectral intercept (SI) parameters. In addition, parametrization of the measured BSC with more complex acoustic scattering models resulted in tumor scattering parameters that include average scatterer diameter (ASD) and average acoustic concentration (AAC) [33–36]. These QUS spectral features have been demonstrated to correlate with patients’ response to NAC both *a priori* and after initiation of chemotherapy [27, 30]. Furthermore, QUS spectroscopy has also been used to monitor therapy response by evaluating the changes in QUS parameters at weeks 1, 4, and 8 after the initiation of NAC with respect to the QUS parameters acquired at week 0 (baseline) [28, 29, 32].

Heterogeneity in tumour micro-environment, physiology and metabolism has shown diagnostic and prognostic usefulness for characterizing cancer [38–42]. Spatial heterogeneities in tumour characteristics have been demonstrated using different imaging modalities, such as magnetic resonance imaging (MRI) [43], positron emission tomography (PET) [44, 45], computerized tomography (CT) [46, 47], and diffuse optical spectroscopy (DOS) [48]. Texture analysis techniques allow quantification of such heterogeneities [49]. In the work here texture analysis methods were as before applied to QUS spectral parametric images, resulting in quantitative textural measures for predicting early patients’ response to NAC.

This study improves upon previous investigations by limiting the number of selected features for developing a response predictive model. Earlier, the model was developed using a combination of nine features from 56 LABC patients [30]. Here, we limit the number of features to approximately 1/10th of the number of observations in each group in order to prevent overfitting [50]. In addition, the model was developed using ultrasound RF data collected from multi-centric cancer centers. This work is a part of ongoing efforts in the inclusion of artificial intelligence tools in diagnostic and predictive models for cancer therapies [25–32]. A necessary step in the use of artificial intelligence tools in diagnostic and prediction is to generalize the model [50]. Consequently, the development of response predictive models from multiples sites provides an indication towards the generalization of the QUS-based framework. This study demonstrates that variations in ultrasound imaging systems, acquisition system settings, and different operators collecting the ultrasound RF data from different sites do not influence the ability of QUS spectral parametric imaging and novel derivative texture methods framework in developing an accurate response predictive model. Findings from this study confirm with results from a recent study that demonstrated that tissue heterogeneity was the dominant feature affecting values of QUS spectral parameters, allowing for the development of a treatment response-monitoring model [32]. Variations in ultrasound system parameters were observed to be smaller than the inherent tissue heterogeneity [32]. These result from the use of a normalization procedure in the QUS spectral analysis that removes instrument-dependent effects [51].

Previous studies have investigated radiomic features of QUS parametric images for predicting patients’ responses prior to treatment initiation [30]. In this study, the pool of radiomic features was extended to include novel texture-derivate features. Texture-derivate features were obtained from a second-pass texture analysis applied to the texture maps of QUS parametric images. These texture maps represent local textural measures of QUS parametric images. These potentially elucidated more distinction between responders and non-responders compared to averaged mean-value and averaged texture features of QUS parametric images. Texture-derivative features have been demonstrated to provide discriminative features in different contexts, for example in the benign versus malignant characterization of breast lesions [25], and in the response prediction of LABC to NAC [31].

In this study, we developed a response predictive model using artificial intelligence tools in order to assess *a priori* response to NAC in patients with LABC. Three standard classification algorithms in the field of machine learning that include linear discriminant analysis (LDA), *k-*nearest neighbours, and support vector machine-radial basis function (SVM-RBF) were evaluated to compare the performance of response predictive models. Model performance was evaluated using leave-one-out cross-validation (LOO-CV). Peak response prediction performance of 84% accuracy and 0.86 AUC was obtained from a combination of features determined from tumour core and tumour rim using the SVM-RBF classification algorithm. The findings here demonstrate the potential of a QUS-based framework as a working tool for early evaluation of breast tumours response to NAC.

## RESULTS

### Patient clinical characteristics

Table 1 summarizes the clinical and pathological characteristics of the patients included in this study. The median age of patients was 51 ± 12 years (range: 27–74 years). The median tumour size in its longest dimension prior to treatment was 3.6 ± 2.5 cm (range: 1.2–11.6 cm). The median tumour size in its longest dimension after treatment was 2.5 ± 3.5 cm (range: 0.0–15.7 cm). Among all the patients, 59 had invasive ductal carcinoma (IDC), and 7 had invasive lobular carcinoma (ILC).

**Table 1:** Patient clinical characteristics

Characteristics	Responder (*n* = 42)	Non-responder (*n* = 32)	Total (*n* = 74)
**Age**: Median ± std (range)	50 ± 12 (27–74)	54 ± 12 (28–71)	51 ± 12 (27–74)
**Histology:**			
**IDC (%)**	81	78	80
**ILC (%)**	7	13	9
**Other (%)**	12	9	11
**ER/PR/HER2:**			
**Triple Negative (%)**	17	22	19
**Non-triple Negative (%)**	83	78	81
**Systemic Treatment:**			
**AC-T (%)**	64	56	61
**FEC-D (%)**	29	41	34
**Other (%)**	7	3	5
**Initial Tumour Size (cm):** Median ± std (range)	3.6 ± 2.5 (1.2–11.6)	3.6 ± 2.4 (1.8–9.8)	3.6 ± 2.5 (1.2–11.6)
**Residual Tumour Size (cm):** Median ± std (range)	1.6 ± 2.2 (0.0–10.0)	3.4 ± 4.2 (1.4–15.7)	2.5 ± 3.5 (0.0–15.7)

Abbreviations: IDC: Infiltrating ductal carcinoma, ILC: Infiltrating lobular carcinoma, ER: Estrogen receptor, PR: Progesterone receptor, FEC-D: 5-fluorouracil, Epirubicin, Cyclophosphamide, and Docetaxel, AC-T: Doxorubicin and Paclitaxel.

The responder group demonstrated a statistically significant (*p* < 0.05) reduction in maximum tumour size from 3.6 ± 2.5 cm prior to treatment compared to 1.6 ± 2.2 cm after treatment. On the other hand, the non-responder group did not show a reduction in maximum tumour size post-treatment compared to pre-treatment status.

Doxorubicin, cyclophosphamide, and paclitaxel (AC-T) chemotherapeutic agents were administered to 61% of the patients. 34% of the patients received 5-fluorouracil, epirubicin, cyclophosphamide, and docetaxel (FEC-D) chemotherapeutic agents. Along with NAC, 30% received trastuzumab in the neoadjuvant setting.

### QUS, texture, and texture-derivate parameters

**Figure 1:**
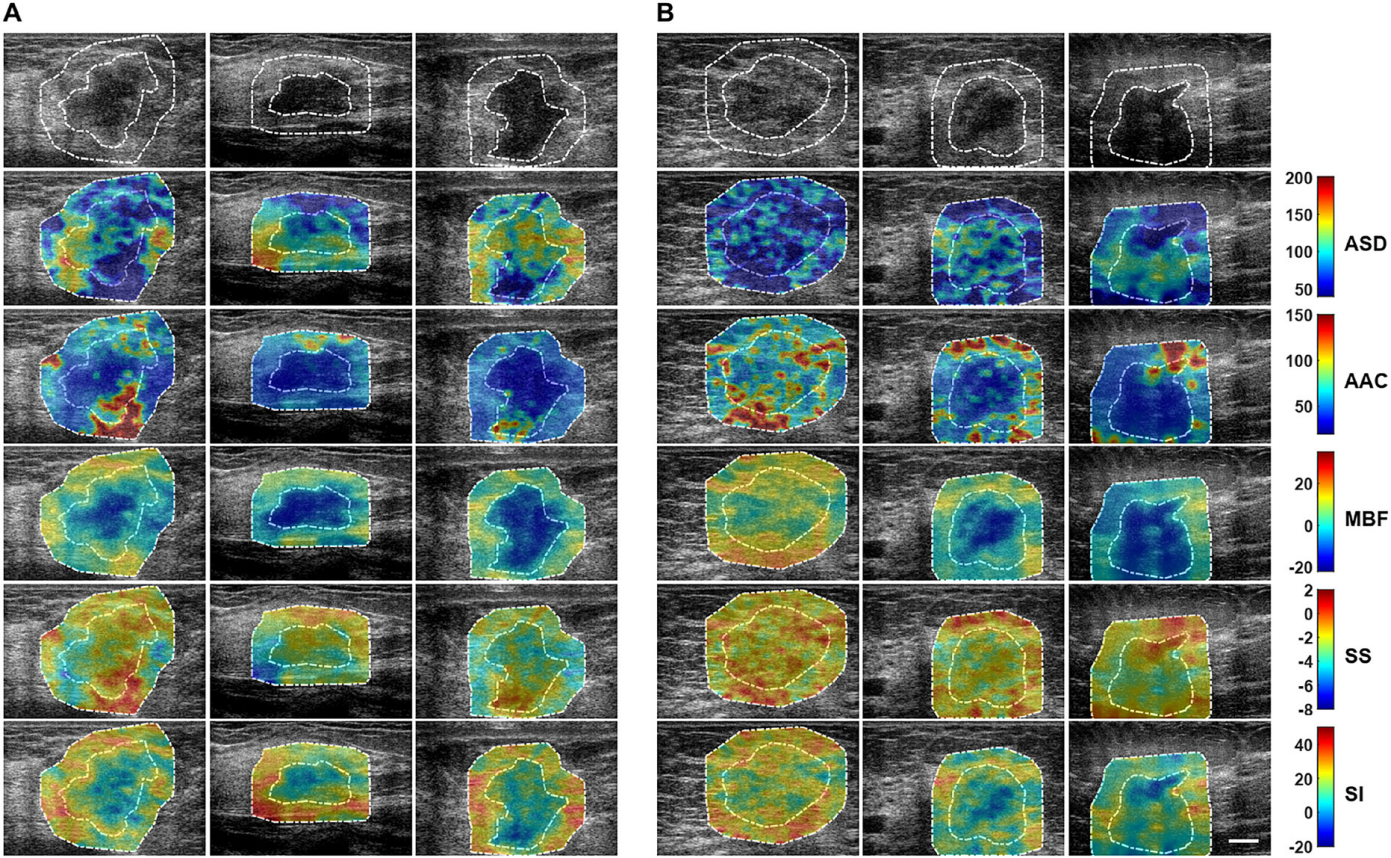
Parametric maps for the two response groups. Representative B-mode US and QUS spectral parametric images of ASD, AAC, MBF, SS, and SI from (**A**) responder (left three columns), and (**B**) non-responder (right three columns) patients with LABC. QUS parametric images include tumour core (central region bounded by closed dotted white curve) and a 5-mm tumour margin (annular region bounded by closed dotted white curves). The colour-bar range is 160 μm for ASD, 130 dB/cm^3^ for AAC, 57 dB for MBF, 10 dB/MHz for SS, and 70 dB for SI. The scale bar represents 1 cm. From these parametric images, mean-value, texture, and image quality features were determined as potential imaging biomarkers for the prediction of response to NAC.

QUS spectroscopy analysis resulted in five QUS parameters that include MBF, SS, SI, ASD, and AAC for classification. Figure 1 presents representative B-mode US images and parametric images of ASD, AAC, MBF, SS, and SI from responder and non-responder patient groups. Tumour B-mode images had a characteristic hypoechoic appearance with parametric maps of QUS features demonstrating greater heterogeneity spatially upon visible inspection.

**Figure 2:**
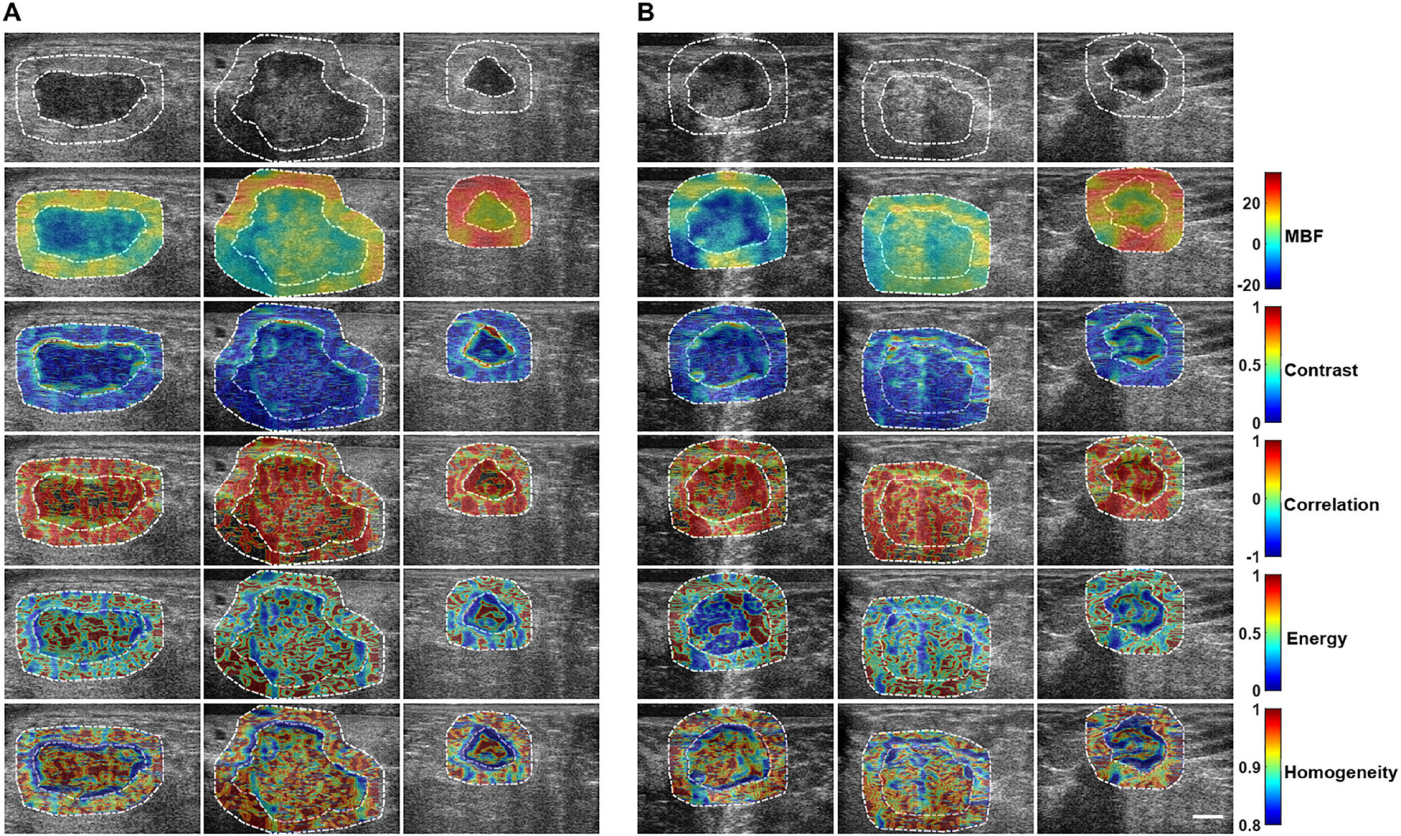
Texture maps for the two response groups. Representative B-mode US images, primary MBF parametric images, and texture-based maps determined from MBF parametric images from (**A**) responder and (**B**) non-responder groups. Texture maps were obtained through the application of sliding window analysis that results in: contrast (MBF-CON), correlation (MBF-COR), energy (MBF-ENE), and homogeneity (MBF-HOM) maps that represent local textural quantification of the primary MBF parametric image. From each of these maps, a second-pass texture analysis was applied to come up with four texture-derivate features that were subsequently used to predict response to NAC and are presented here.

In addition, intra- and peri-tumoural heterogeneities were quantified using texture and novel derivative texture methods. Representative QUS parameters and their associated texture features are presented in Supplementary Figure 1. Not all features were statistically significantly different between the responder and on-responder groups. Figure 2 presents representative texture maps of the mid-band fit and related texture-based parametric images from responder and non-responder patient groups. These texture maps represent local textural quantification of QUS parametric images. The contrast, homogeneity and energy texture-features appeared to demonstrate the most significant change at the interfaces between tumour core and rim regions.

Texture analysis of these texture maps resulted in texture-derivate features that potentially better separate the two groups. Representative results are presented in Supplementary Figure 2 for one QUS parameter parametric-map texture-derivate (texture-of-texture) feature set. Supplementary Figures 3–16 show all texture-based, texture-derivate-based parameters, and image quality features considered in this study.

### Comparison of classification algorithms

**Figure 3:**
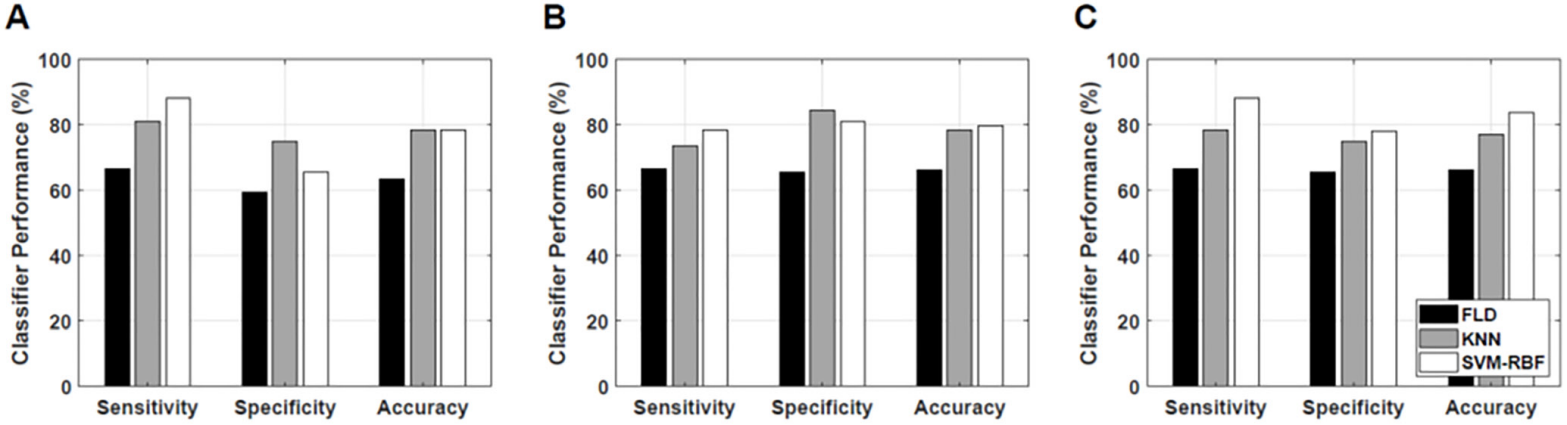
Classification results. Classification performance of the response prediction model from different classification algorithms using features determined from (**A**) tumour core, (**B**) 5-mm tumour margin, and (**C**) both core and 5-mm margin.

Figure 3 presents bar plots of classification results: sensitivity, specificity, and accuracy of response predictive model developed utilizing tumour core regions, tumour rim regions, and a combination of tumour core and rim analysis, respectively using different classification algorithms. The best performance was attained from a model utilizing combined features from the tumour core and a 5-mm tumour margin using an SVM-RBF classification algorithm.

Table 2 tabulates response prediction results from tumour core analysis using different classification algorithms. Using a nonlinear algorithm (SVM-RBF) achieved the best classification performance of 78% accuracy and 0.79 AUC. Table 3 tabulates response prediction results using rim analysis using different classification algorithms. A response predictive model developed using features extracted from tumour rim achieved a peak performance of 80% accuracy and a 0.82 AUC, using an SVM-RBF classification algorithm. Table 4 tabulates response prediction results from a combined core and rim analysis using different classification algorithms. The best response prediction results of 84% accuracy and 0.86 AUC were achieved using the SVM-RBF classification algorithm. A combination of features extracted from the tumour core and tumour margin resulted in a response predictive model that best separate responders from non-responders.

**Table 2:** Classification results of response predictive model using features from tumour core

Classification Algorithm	Sensitivity	Specificity	Accuracy	AUC	PPV	NPV
**LDA**	67%	59%	64%	0.58	68%	58%
**KNN**	81%	75%	78%	0.78	81%	75%
**SVM-RBF**	88%	66%	78%	0.79	77%	81%

**Table 3:** Classification results of response predictive model using features from tumour margin

Classification Algorithm	Sensitivity	Specificity	Accuracy	AUC	PPV	NPV
**LDA**	67%	66%	66%	0.60	72%	60%
**KNN**	74%	84%	78%	0.72	86%	71%
**SVM-RBF**	79%	81%	80%	0.82	85%	74%

**Table 4:** Classification results of response predictive model using features from both tumour core and tumour margin

Classification Algorithm	Sensitivity	Specificity	Accuracy	AUC	PPV	NPV
**LDA**	67%	66%	66%	0.60	72%	60%
**KNN**	79%	75%	77%	0.74	80%	73%
**SVM-RBF**	88%	78%	84%	0.86	84%	83%

## DISCUSSION

The work here is a radiomics study of QUS spectral parametric imaging using artificial intelligence tools for the *a priori* prediction of response to NAC in 74 patients with LABC. In contrast to previous studies, the work here improved study design by limiting the number of selected features in developing a response predictive model in order to prevent overfitting [50]. In addition, novel texture-derivative features of QUS spectral parametric images were utilized to build a robust response predictive model. Furthermore, the model was developed and evaluated using ultrasound RF data that were acquired from multiple cancer institutions that include Sunnybrook Health Sciences Center (SHSC), MD Anderson Cancer Center (MD-ACC), and St. Michaels Hospital (SMH). This study represents an ongoing effort towards building and validating a response predictive model using an external data set in order to assess the generalizability of the developed model (geographical validation) [50].

Mean-value, texture, and texture-derivate features were determined from QUS spectral parametric images that include tumour core and a 5-mm tumour margin. The margin size was selected based on previous investigations, where it was changed from 0.3 to 1.0 cm [30]. The consequent tumour core and margin features were used to develop a multi-feature response predictive model that classifies responders (‘R’) from non-responders (‘NR’) in advance of the initiation of NAC. Ground truth clinical response was determined using a modified RECIST methodology that considers primarily the change in tumour size between “pre-treatment” and “post-treatment” standard clinical diagnostic imaging, with the addition of tumour cellularity assessment from post-surgical histopathology. In order to build a robust response predictive model, three standard classification algorithms in the field of machine learning were evaluated. These included linear discriminant analysis (LDA), *k-*nearest neighbours (KNN), and a support vector machines-radial basis function (SVM-RBF). The performance of each classification algorithm was assessed objectively using the ROC analysis, providing metrics of sensitivity, specificity, accuracy, AUC, PPV, and NPV. The study here demonstrated that a combination of four features determined from tumour core and a 5-mm tumour margin resulted in an accurate response predictive model with 88% sensitivity, 78% specificity, 84% accuracy, 0.86 AUC, 84% PPV, and 83% NPV, using an SVM-RBF classification algorithm. Model development from multi-centric data provided an indication of the potential of the QUS spectroscopy and texture analysis approaches towards their generalization.

As mean-values of QUS spectral parametric images do not provide information about image heterogeneity, texture methods were used to quantify tumour heterogeneities, which were evident in parametric images. Both texture and texture-derivate analyses were carried out. Texture-derivate features resulted from the creation of intermediary texture maps of QUS parametric images, followed by a second-pass texture analysis [25, 31]. Texture-derivate analysis quantified local textural variations of QUS parametric images that were more sensitive to predict response compared to that using mean-value and texture features alone. Texture-derivate features have been demonstrated recently to contribute to hybrid biomarkers for the characterization of breast lesions [25] and for the response prediction of LABC patients to NAC [31].

Margin analysis akin to that in this study has been demonstrated earlier to have sufficient discriminative power to differentiate responders from non-responders [30]. In addition, margin analysis has also been explored in the characterization of breast lesions [25, 26]. Tumour responses to NAC are thought to be different between responders and non-responders. Histopathological analysis has shown that the tumour margin consists of microscopic infiltration from the primary tumour into the surrounding normal breast tissues [52, 53]. This makes rim analysis of QUS spectral parametric images potentially useful for predicting early treatment response.

In this study, a response predictive model developed using features determined from tumour core achieved the best response prediction of 88% sensitivity, 66% specificity, 78% accuracy, 0.79 AUC, 77% PPV, and 81% NPV using an SVM-RBF classification algorithm. Using features extracted from the tumour margin, a peak response prediction performance of 78% sensitivity, 81% specificity, 80% accuracy, 0.82 AUC, 85% PPV, and 74% NPV was obtained using an SVM-RBF classification algorithm. Results indicated that the response predictive model developed using rim information alone achieved better prediction results compared to that developed using core information alone, with accuracies of 80% versus 78% and AUCs of 0.82 versus 0.79, respectively. Furthermore, the combination of discriminating features from core and margin resulted in a more robust response prediction performance with 88% sensitivity, 78% specificity, 84% accuracy, 0.86 AUC, 84% PPV, and 83% NPV, also achieved using an SVM-RBF classification algorithm. The optimum feature set included CMR-SI, Core-SS-ENE-CON, Core-ASD-HOM-CON, and Margin-ASD-ENE-COR features. Texture-derivate features dominated the optimum response predictive model.

Among the classification algorithms evaluated, the SVM-RBF performed the best-utilizing features from tumour core only, tumour margin only, and the combination of tumour core and margin. Using a KNN classification algorithm, the best response prediction of 81% sensitivity, 75% specificity, 78% accuracy, 0.77 AUC, 81% PPV, and 75% NPV was achieved utilizing core information only. Among the standard classification algorithms evaluated, linear discriminant analysis performed the poorest. This can be attributed to the fact that a linear classification algorithm works best only for linearly separable data. Nonlinear classification algorithms in the SVM-RBF methodology outperformed the other two classification algorithms as has been demonstrated previously in various contexts [25, 26, 29].

Previously, *a priori* response to NAC has been evaluated in 56 patients with LABC [30]. In that study, the best response prediction of 90% sensitivity, 79% specificity, 88% accuracy, and 0.81 AUC with a KNN classification algorithm was achieved using mean-values and texture features extracted from both tumour core and tumour margins [30]. This resulted from a combination of nine features obtained from sequential feature selection [30]. However, the number of features used in that earlier study is likely to introduce overfitting of the developed response predictive model [50]. The current study design improved this aspect by limiting the number of selected features for building the response predictive model to a total of only four features. Furthermore, the pool of radiomic features was extended through the inclusion of texture-derivate features. Texture derivative method has demonstrated its utility in the characterization of breast lesions [25] and early prediction of therapy responses to NAC [31]. In that study, Dasgupta *et al.* developed a response predictive model that achieves the best 87% sensitivity, 81% specificity, 82% accuracy, and 0.86 AUC in predicting responders from non-responders prior to initiation of NAC [31]. In contrast to the work presented herein, the response predictive model developed by Dasgupta *et al.* consisted of 100 LABC patients’ data acquired from a single institution [31]. The development of response predictive model from data collected from multi institutions and acquired by different operators and using different ultrasound system settings indicates the robustness of the QUS framework. Recently, Sannachi *et al.* demonstrated the robustness of a response-monitoring model with respect to variations in ultrasound system parameters [32]. Their study identified tissue heterogeneity as the dominant feature causing variations in QUS spectral and texture parameters obtained from other clinical ultrasound systems [32]. Proper normalization procedures result in comparable QUS and texture-based parameters from other clinical ultrasound systems and different acquisition settings implemented by different operators [32]. This permits for the development of *a priori* response predictive model based on those features, as demonstrated herein. Recently, DiCenzo *et al.* also reported a multi-institution response predictive model utilizing mean-values and texture features of QUS spectral parametric images from the tumour core [54]. In that study, the best response predictive results of 91% sensitivity, 83% specificity, 87% accuracy, and 0.73 AUC were attained [54]. The work presented herein expanded this previous analysis through the addition of tumour margin analysis and the inclusion of texture-derivate features. In addition, we also implemented stricter data inclusion criteria that result in the exclusion of US RF data from a single site. Although, the best selected features in this study do not demonstrate *p* < 0.05 statistical significant differences when assessed individually as in [54], a combination of some of discriminating features still resulted in a multi-feature classification model that predict *a priori* response to NAC with 88% sensitivity, 78% specificity, 84% accuracy, and 0.86 AUC. In contrast to the results in [54] where the best classification algorithm was the instance-based nearest neighbours, here the SVM-RBF results in the best response prediction model by combining features from both tumour core and tumour margin. Nonlinear classification algorithm in SVM-RBF proves to be more robust in separating responders from non-responders.

*A priori* response predictive model to NAC has also been explored using texture assessment of images obtained from different imaging modalities. Tran *et al.* developed a response predictive model using textural features extracted from pre-treatment diffuse optical spectroscopy (DOS) functional maps [55]. In their study, univariate parameter of homogeneity of HbO_2_ map achieved the best 86% sensitivity, 89% specificity, and 88% accuracy from 37 LABC patients undergoing NAC. Recently, Moghadas-Dasterdji *et al.* used quantitative textural features extracted from computerized tomography (CT) images for developing *a priori* response predictive model using different machine learning classification algorithms [56]. Their study included 72 LABC patients undergoing NAC and obtained the best classification results of 80% sensitivity, 88% specificity, 84% accuracy, and 0.89 AUC_0.632+_ using an Adaboost decision tree (DT) classification algorithm [56]. The response prediction results of these different imaging modalities are comparable with the results presented in this study.

QUS-based response predictive models offer oncologists useful imaging tools for potentially enabling adaptive chemotherapy for patients with LABC. The types of chemotherapeutic drugs administered to specific patients potentially can be tailored depending on whether a patient is predicted to be a responder or non-responder based on QUS-texture-based analyses prediction. Furthermore, different QUS-based response predictive models can be used to monitor treatment response to NAC in LABC patients over the course of chemotherapy. In that setting, using a modified approach the chemotherapeutic agents can be customized based on patient responses to treatment. If a patient is determined to be non-responsive after initial treatment or at a particular week based on QUS, the oncologist has an option to switch the treatment plan to different chemotherapeutic drugs (‘chemo-switch’) that are potentially more helpful rather than subjecting the patient to systemic agents that are not demonstrating treatment effect.

## MATERIALS AND METHODS

### Study design

A total of 74 patients (42 responders and 32 non-responders) with LABC undergoing NAC were enrolled in this study. The cohort consisted of patients accrued from three different sites: Sunnybrook Health Sciences Center (SHSC) (*n* = 50), University of Texas MD Anderson Cancer Center (MD-ACC) (*n* = 23), and St. Michaels Hospital (SMH) (*n* = 1). The study was performed in accordance with institutional research ethics guidelines at each respective site. Patients were included in this study after obtaining written informed consent. Magnetic resonance images (MRI) were acquired as a part of the treatment in order to determine pre-treatment tumour size. Core needle biopsy specimens were collected from all patients prior to the start of NAC in order to obtain histological confirmation, determine tumour subtype and hormone receptor status consisting of estrogen receptor (ER), progesterone receptor (PR), and HER2 expression. A full course of NAC that lasted typically for 4–6 months was completed by all patients. Subsequently, these patients underwent either lumpectomy or mastectomy. A board-certified pathologist examined the specimens using whole-mount 5” by 7” pathology slides digitized using a confocal scanner (TISSUEscope^™^, Huron Technologies, Waterloo, ON, Canada). Clinical/pathological tumour response was determined at the end of their treatment using a modified response (MR) grading system. After surgery, adjuvant therapies that consist of radiation, maintenance Transtuzumab for HER2 positive tumours or endocrine therapy (for hormonal-receptor positive tumours) were commenced as per standard institutional practice [30].

The MR grading system was based on a combination of histopathological evaluation (of residual tumour cellularity) and the Response Evaluation Criteria in Solid Tumour (RECIST). The RECIST-based change was defined as the percent change of tumour size (in its longest dimension) between pre-treatment and post-treatment times. Magnetic resonance imaging scans were used to determine these sizes as part of patients’ standard of care. MR score of 1 was assigned if there is no reduction in tumour size. MR score of 2 was assigned if there is a diminishment of up to 30% in tumour size. MR score of 3 was assigned if the reduction in tumour size is between 30–90%. MR score of 4 was assigned for diminishment of more than 90% in tumour size. Lastly, a MR score of 5 was assigned if there was no evidence of residual tumour at all. In addition to these RECIST-based criteria, residual tumour cellularity was also considered for evaluating response. Residual tumour cellularity alone was introduced elsewhere in a new histological grading system to assess breast cancers’ response to NAC and had demonstrated a potential in predicting overall survival and disease-free intervals [5, 6]. In this study, a threshold of 5% tumour cellularity was chosen. Tumour with residual cellularity less than or equal to 5% (<= 5%) were deemed a responder, otherwise tumours were a non-responder based on cellularity criterion alone. The overall response combined both RECIST-based criteria in tumour size reduction and residual tumour cellularity. A patient was a responder (‘R’) if either the reduction in tumour size was greater than 30% or residual tumour cellularity was low (<= 5%). A patient was a non-responder (‘NR’) if there was a reduction in tumour size that was less than 30%, or there was an increase in tumour size, and there was a high residual tumour cellularity (> 5%). These were used as the ground truth in the binary classification of response.

### Quantitative ultrasound and texture parameter estimation

Ultrasound RF data were collected prior to the start of NAC using a Sonix RP (Analogic Medical Corp., Vancouver, Canada) or a GE-LOGIQ E9 (GE Healthcare, Milwaukee, Wisconsin, USA) clinical ultrasound imaging system. The RP system was equipped with a linear array transducer operating at 6.5 MHz center frequency with a bandwidth of 3–8 MHz. The GE system was equipped with a 9L-D linear array transducer, operating at 6.0 MHz center frequency and a bandwidth of 3.5–8.5 MHz. Beamformed RF data were acquired using a 40 MHz sampling rate.

We performed QUS spectral analysis over ROIs that include tumour core and a 5-mm margin. A sliding window analysis using a 2-mm by 2-mm kernel was used to create parametric images of QUS spectral parameters. The size of the window was chosen to include a sufficient number of acoustic wavelengths to ensure reliable spectral estimation, while preserving image texture. A 94% window overlap was used between adjacent windows both axially and laterally.

A Hanning gating function was applied on individual RF scan lines within the window along the range direction for spectral analysis. The power spectrum of the sample was estimated using the Fast Fourier Transform (FFT) technique. Several independent adjacent RF signals within the window were used to obtain an averaged power spectrum that better represent the true power spectrum of the sample. The normalized power spectrum was obtained using a reference phantom technique [51, 57]. The reference phantom was composed of 5–30 μm glass beads embedded in a homogeneous medium of oil droplets that were immersed in gelatin. The measured attenuation coefficient and speed of sound of the phantom were 0.8 dB/cm/MHz and 1,540 m/s, respectively (University of Wisconsin, Department of Medical Physics, Madison, WI, USA).

Attenuation correction was performed in order to compensate for attenuation due to propagation of ultrasound through intervening tissue layers. An assumed attenuation coefficient of 1 dB/cm/MHz for the overlying breast tissues was used [58, 59]. The tumour attenuation coefficient was estimated (ACE) using a spectral difference method by comparing the rate of change in the log spectral power magnitude with depth (over the tumour region) of the sample relative to the reference phantom for each frequency within the frequency bandwidth [57]. The window and the ROI axial length sizes conformed to the recommendations specified by Labyed *et al.* [57] for accurate and precise attenuation coefficient estimation using a spectral difference method on clinical linear array ultrasound system. MBF, SS, and SI spectral parameters were obtained from linear parametrization of the attenuation-corrected NPS over the frequency bandwidth. MBF and SI parameters correlate with the size, concentration, and relative acoustic impedance of acoustic scatterers, while SS parameter depends on the size of acoustic scatterers [35]. Subsequently, the measured backscatter coefficient (BSC) can be estimated via [35],

(1) $$\sigma_{m}(f)=\sigma_{r}(f)\frac{{\left|S_{m}(f)\right|}^{2}}{{\left|S_{r}(f)\right|}^{2}}e^{\left\{4(\alpha_{m}-\alpha_{r})\left(R+\frac{\Delta z}{2}\right)\right\}},$$

where *S_m_*(*f*) and *S_r_*(*f*) are the RF spectra from the sample and the reference phantom, respectively. Variables α_*m*_ and α_*r*_ represent the attenuation functions from the sample and the reference phantom, respectively. Parameter R is the distance from the transducer face to the proximal side of the ROI window, and Δz is the window length. Average scatterer diameter (ASD) *a_eff_* and average scatterer concentration (AAC) *n*_z_ parameters can be estimated through the fitting of a theoretical BSC from spherical Gaussian form factor model σ_*theor*_(*f*) to the measured BSC [33–36]. The theoretical BSC is expressed as [33–36]

(2) $$\sigma_{theor}(f)=Cf^{4}a_{eff}^{6}\bar{n}\gamma_{0}^{2}F(f,a_{eff}),$$

where $C=\frac{\pi^{2}}{36c_{l}^{4}}$ and *c_l_* is the speed of sound. *F* (*f*, *a_eff_*) is the form factor that describes frequency-dependent backscattering. The AAC represented the net scattering strength [33–36]. It is defined as the product of average number density of scatterers $\bar{n}$ and squared of the fractional difference in the acoustic impedance between a scatterer and the surrounding medium $\gamma_{0}^{2}$ [33–36].

The sliding window was moved across each point in the ROI, resulting in parametric images of mid-band fit, spectral slope, spectral intercept, average scatterer diameter, and average acoustic concentration. Mean-values from these parametric images were obtained from tumour core and a 5-mm tumour margin ROIs and subsequently used as potential discriminating features to build a response predictive model.

In addition to mean-values, texture and derivative texture methods were used to quantify spatial heterogeneity of QUS parametric images. We performed texture analysis using a gray level co-occurrence matrix (GLCM) method to quantify intra-and peri-tumour heterogeneities. The GLCM realizes second-order statistical analysis by studying the spatial relationship between neighboring pixels in an image [49]. The full range of gray level intensities in each parametric image was linearly scaled into 16 discrete gray levels. Symmetric GLCM matrices were created from each parametric image at inter-pixel distances: 1, 2, 3, 4, 5 pixels and at four angular directions: 0°, 45°, 90°, and 135°. From these GLCM matrices, textural features of contrast, correlation, energy, and homogeneity were extracted and subsequently averaged over distances and angular directions:

(3) $$Contrast=\sum_{i,j=0}^{N_{g}}{\left|i-j\right|}^{2}p(i,j)$$

(4) $$Correlation=\frac{1}{\sigma_{i}\sigma_{j}}\sum_{i,j=0}^{N_{g}}\left(i-\mu_{i})(j-\mu_{j})p(i,j\right)$$

(5) $$Energy=\sum_{i,j=0}^{N_{g}}p^{2}(i,j)$$

(6) $$Homogeneity=\sum_{i,j=0}^{N_{g}}\frac{p(i,j)}{1+\left|i-j\right|}$$

In equations. 3, 4, 5, and 6, the *p*(*i, j*) is the probability of having neighboring pixels of intensities *i* and *j* in the image, and *N_g_* denotes the number of gray levels. Contrast quantifies local gray-level variations in an image. Smoother image features produce a lower contrast, while coarser image features result in higher contrast. Correlation represents the linear correlation between neighbouring pixels. Energy measures textural uniformity between neighboring pixels. Homogeneity quantifies the incidence of pixel pairs of different intensities.

Texture-derivate analysis was subsequently applied to the parametric images. In contrast with a previous texture analysis approach that produced averaged texture measures, texture-derivate analysis was carried out through the creation of intermediary texture-encoded maps using a 15-pixel by 15-pixel window that corresponds to 0.27 mm axially and 0.23 mm laterally. Each pixel in these texture-encoded maps represents the quantification of local textures across the 15-pixel by 15-pixel window. Therefore, the GLCM matrix for the construction of texture maps only considers inter-pixel distance of 1. A second pass texture analysis was subsequently performed on these texture maps, resulting in texture-derivate features. Texture-derivate features provided textural assessment of the texture maps obtained from QUS parametric images.

Analyses included tumour core and a 5-mm margin, and image quality metrics consisting of a core-to-margin ratio (CMR) and core-to-margin-contrast ratio (CMCR) as a manner of comparing pixel intensities between the two regions:

(7) $$CMR=\frac{mean(ROI_{Core})}{std(ROI_{Margin})}$$

(8) $$CMCR=\frac{\left|mean(ROI_{Core})-mean(ROI_{M\arg in})\right|}{\frac{1}{2}\left(std(ROI_{Core})+std(ROI_{Margin})\right)}$$

These two parameters have been used as potential imaging biomarkers for assessing treatment response [30]. CMR compares the level of desired signal to the background noise [30]. CMCR is like CMR but also considers bias in an image [30].

### Classification algorithms

Mean-value, texture, and texture-derivate features for tumours were estimated from each scan plane and subsequently averaged over all scan planes. These weighted averaged measures were used for building a response predictive model. Feature selection was performed using forward sequential-feature-selection (SFS). F1-score (the harmonic mean of precision and recall) was used as the performance metric for measuring accuracy. A response predictive model was developed using a combination of four features that best predicted patient response as either a responder or non-responder. The number of features was chosen to limit overfitting in which a predictive model customizes itself too much to the training data and limits its ability to generalize to new data [50]. Leave-one-out cross-validation (LOO-CV) was used to develop and evaluate the performance of the response predictive model. Leave-one-out cross-validation (LOO-CV) involved training the classification model with all observations except one while the left-out observation was used for testing the developed model. The process was repeated until all observations are left out for testing at least once.

In order to develop a highly accurate response predictive model, three standard computational algorithms in the field of machine learning were evaluated including LDA, KNN, and SVM-RBF. The performance of these algorithms was assessed using the receiver operating characteristics (ROC) analysis, providing sensitivity, specificity, accuracy, and AUC (area under the ROC curve), positive predictive value (PPV), and negative predictive value (NPV) metrics. LDA using probabilistic generative models can be described as estimating the posterior probability of assigning an input vector x into one of the two classes by assuming that probability density function of each class is a Gaussian [60]. A linear classifier works optimally for linearly separable data. The KNN method is an instance-based classification algorithm that was used to predicts class association of a test point in the feature space based on most of the points neighbouring the test point and the distance between those points to the test point. The KNN classifier used *k* = 1, 3, 5 nearest neighbours. The SVM-RBF is a nonlinear classification algorithm that maximizes the margin between the two classes and predicts class association of the test data based on which side of the margin they fall [60–62]. Kernel functions are used to map the input data into a higher-dimensional space where the data are supposed to have better distribution, prior to selecting an optimal separating hyperplane in this higher-dimensional feature space. A Gaussian radial basis function (RBF) was used as the kernel function in this study. The kernel function used the soft margin parameter C and the free parameter γ. These kernel parameters were optimized using a grid search method.

Classification using either features from the core or the margin utilized 105 features. These include 5 mean-value, 20 texture, and 80 texture-derivate features from five parametric images. Classification using features from both the core and the margin included 220 features comprising of 105 features from the core, 105 similar features from the margin, and 10 image quality features.

## CONCLUSIONS

This study demonstrates a working *a priori* response predictive model (84% accuracy and 0.86 AUC), developed using QUS and texture-derivate parameters that were determined from US RF data obtained from multiple cancer centers. Model development using multi-centric data provided an indication towards generalizing the QUS framework. This work paves the way towards an implementation of QUS and texture analyses imaging tools for oncologists to provide customized cancer treatment adjusted to specific patient.

## SUPPLEMENTARY MATERIALS


