# Supplementary material for: A priori prediction of response in multicentre locally advanced breast cancer (LABC) patients using quantitative ultrasound and derivative texture methods
Source: Oncotarget. 2021 Jan 19;12(2):81–94. doi: 10.18632/oncotarget.27867 (PMC7825636; doi:10.18632/oncotarget.27867)
Supplement: Supplementary file 1 [file oncotarget-12-81-s001.pdf]

A priori prediction of response in multicentre locally advanced breast cancer (LABC) patients using quantitative ultrasound and derivative texture methods

SUPPLEMENTARY MATERIALS

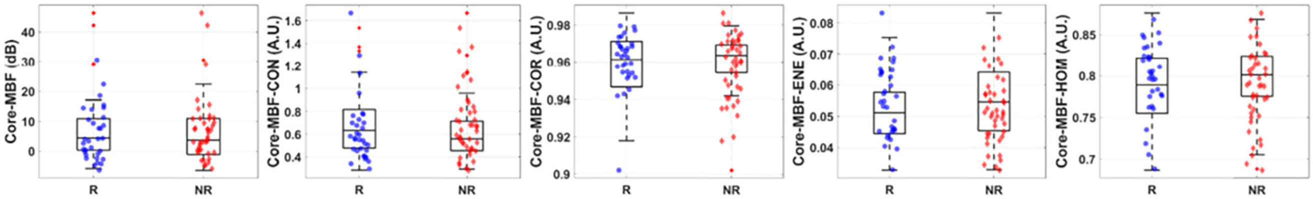

Supplementary Figure 1: Representative box and scatter plots of mean-value and texture features (CON: contrast, COR: correlation, ENE: energy, and HOM: homogeneity) of MBF QUS parameter from core ROI between responder ('R') and non-responder ('NR') groups.

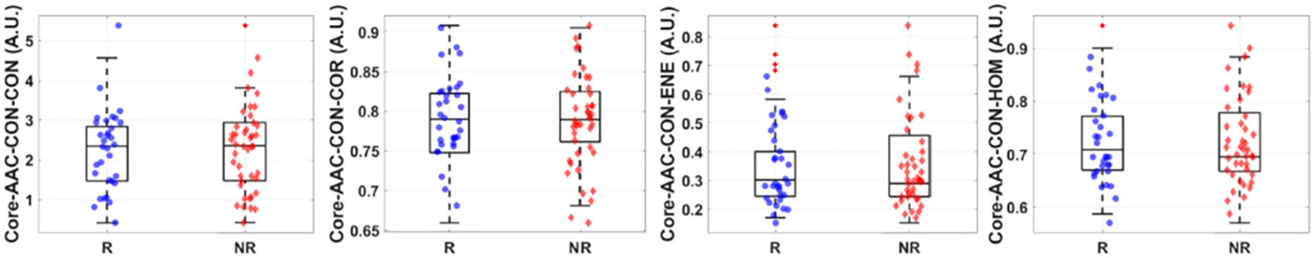

Supplementary Figure 2: Representative box and scatter plots of texture-derivate features of Core-AAC-CON texture feature (contrast image of AAC parametric image from core ROI) between responder ('R') and non-responder ('NR') groups.

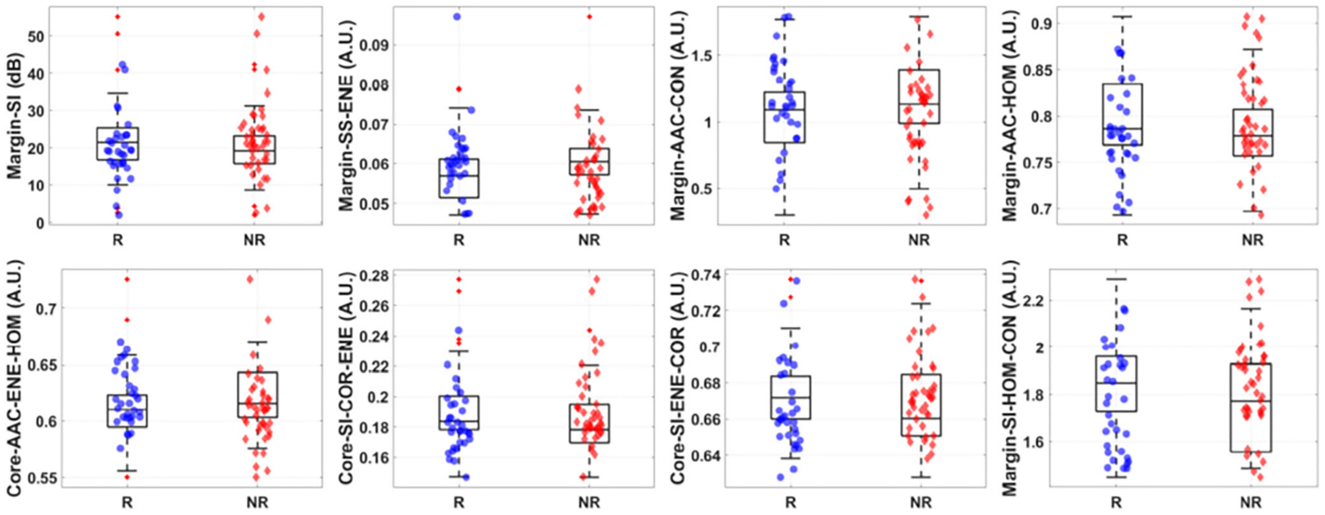

Supplementary Figure 3: Representative box and scatter plots of mean-value, texture, and texture-derivate features that demonstrate the most statistical significant difference between responder ('R') and non-responder ('NR') groups. The upper row shows mean-value and texture features from tumor margin. The lower row depicts texture-derivate features from both tumor core and tumor margin that provide the most discriminative power. There are 220 features from tumor core and 5-mm margin, including 10 image quality features, available for feature selection.

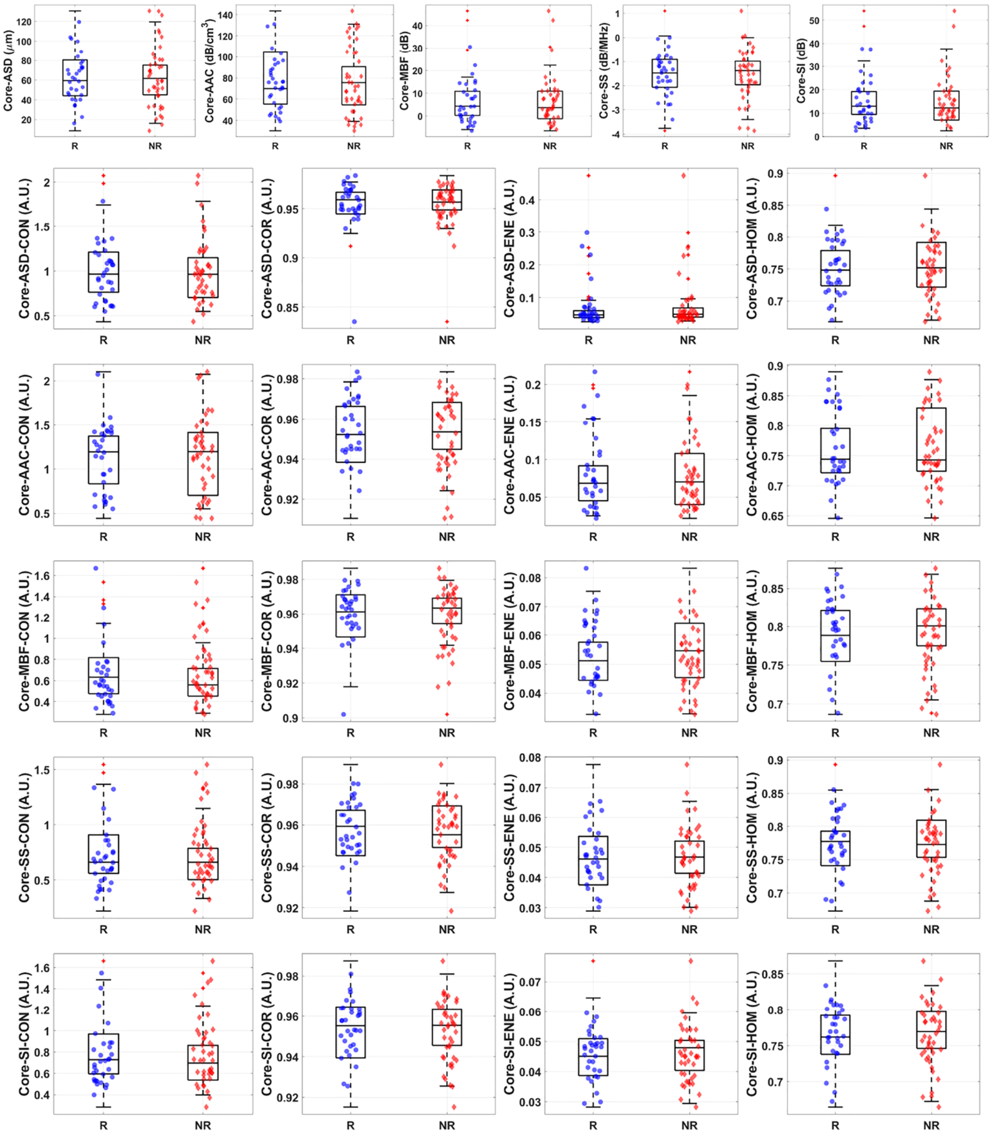

Supplementary Figure 4: Mean-values and texture features of core ROI.

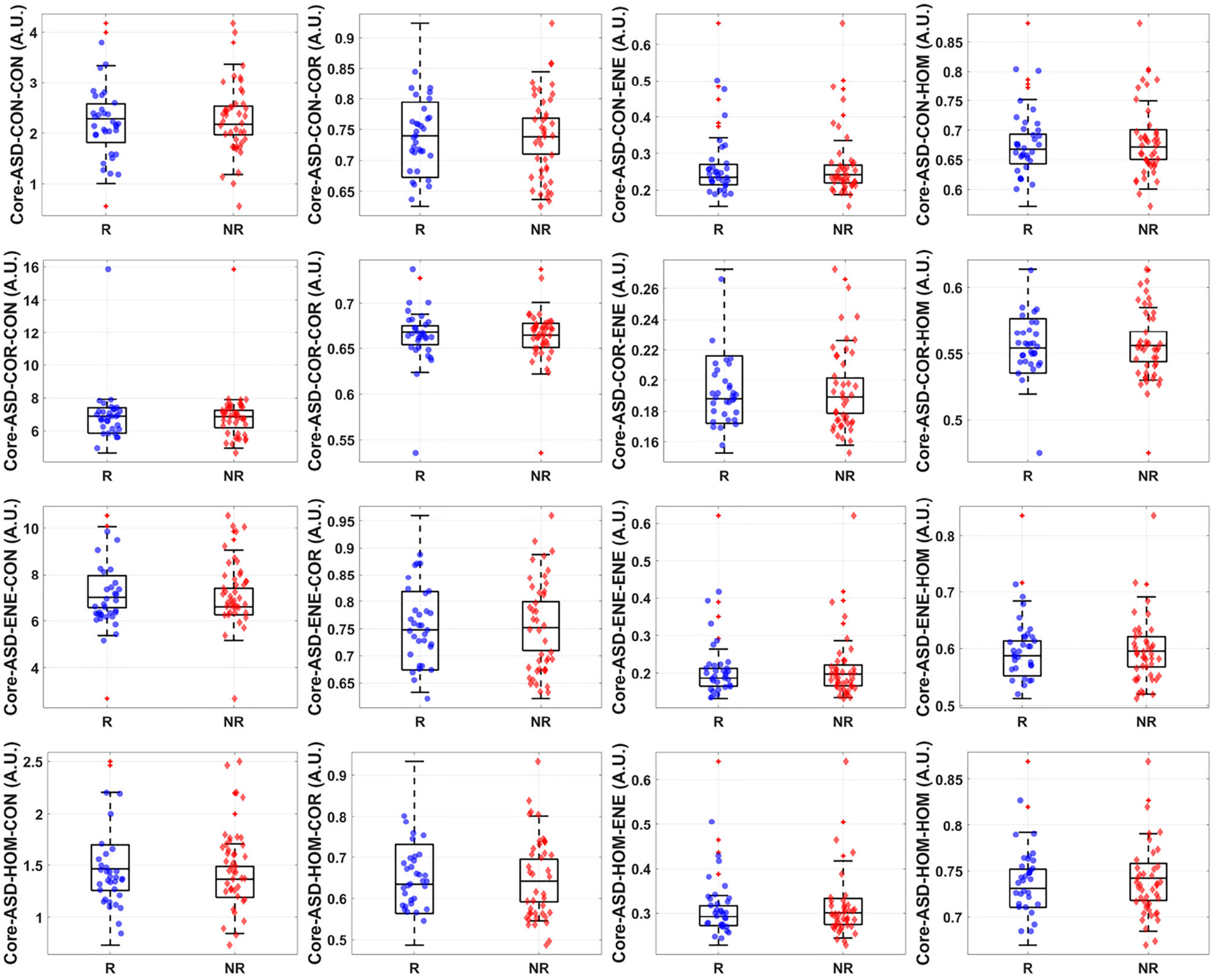

Supplementary Figure 5: ASD Texture-derivate features of core ROI.

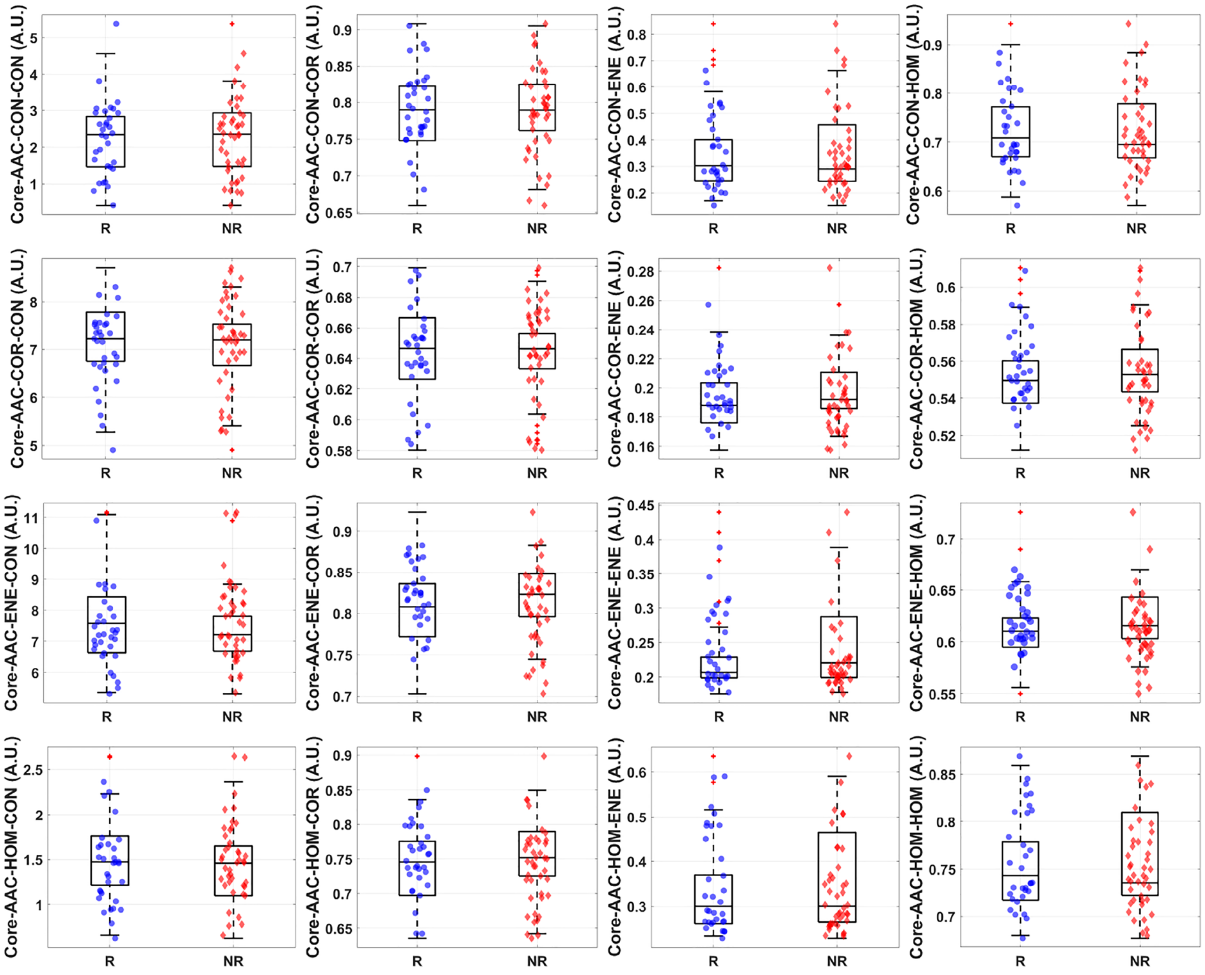

Supplementary Figure 6: AAC Texture-derivate features of core ROI.

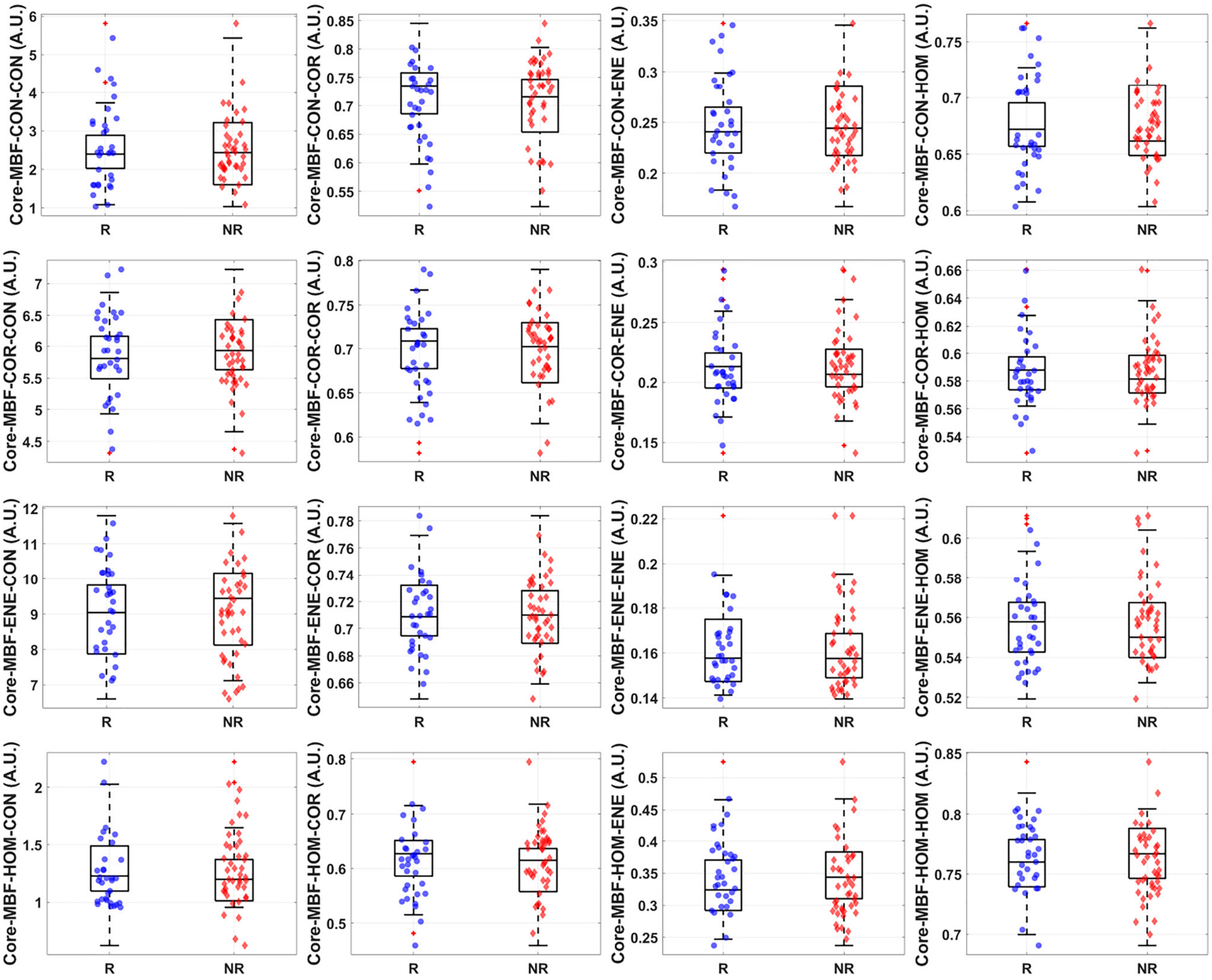

Supplementary Figure 7: MBF Texture-derivate features of core ROI.

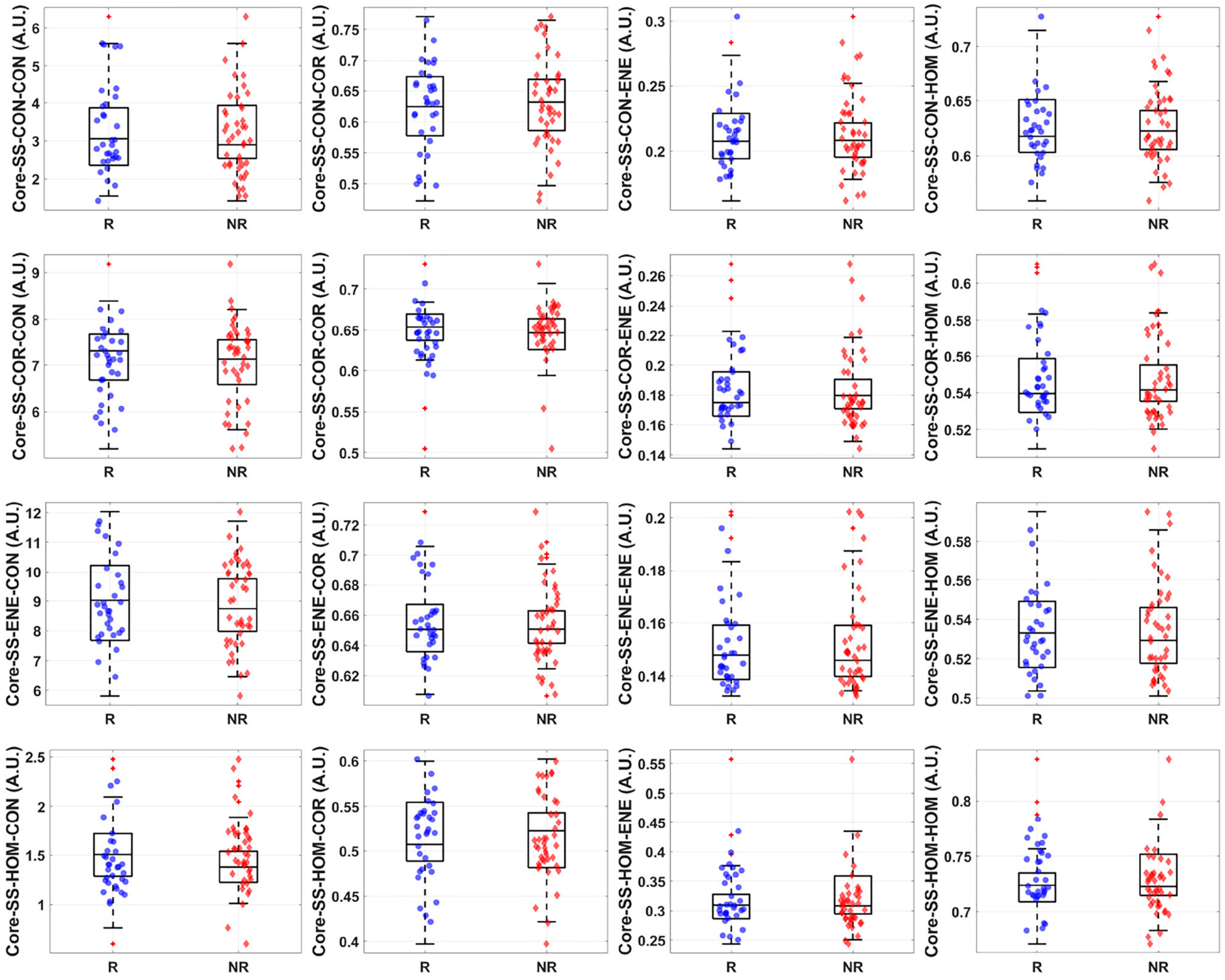

Supplementary Figure 8: SS Texture-derivate features of core ROI.

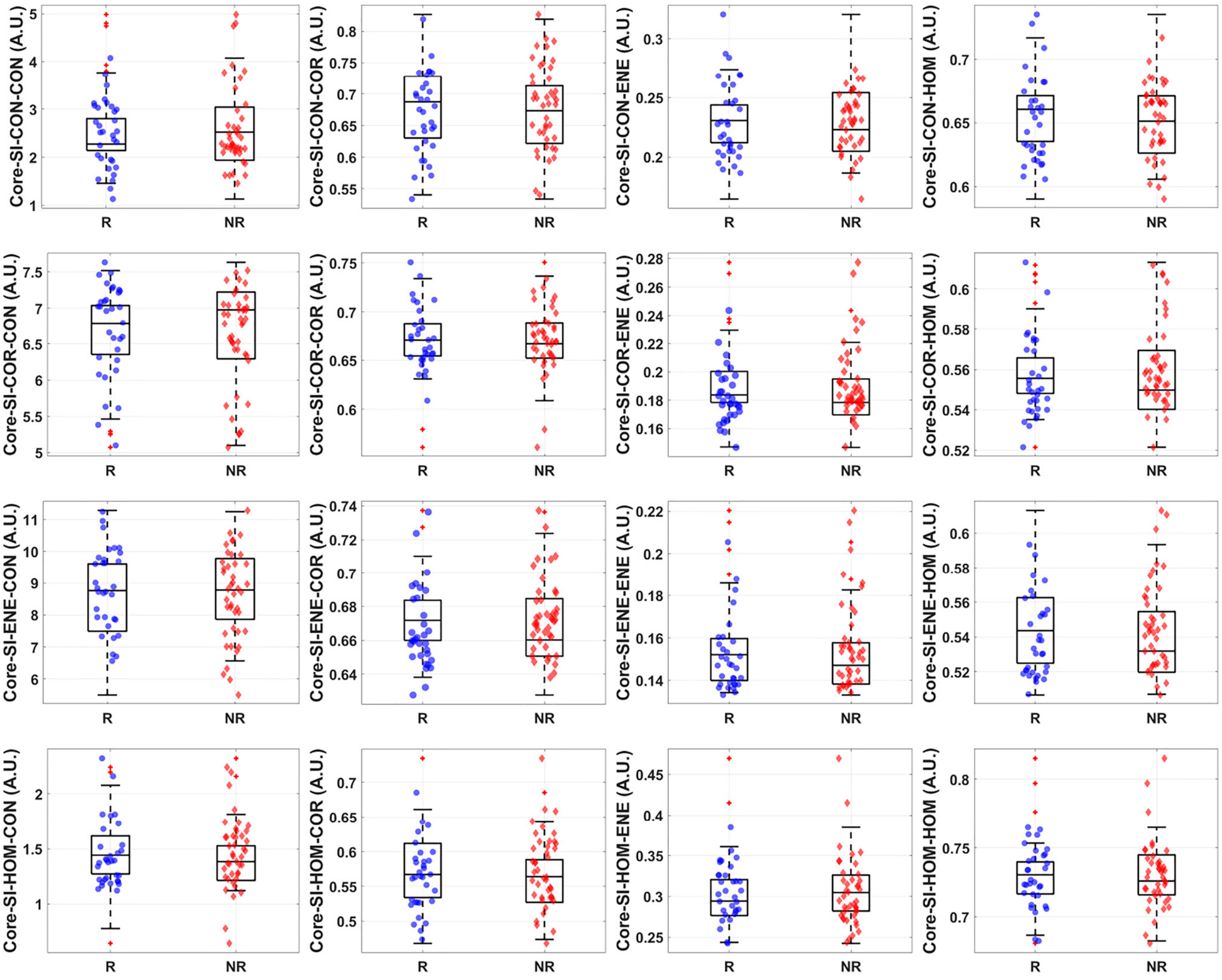

Supplementary Figure 9: SI Texture-derivate features of core ROI.

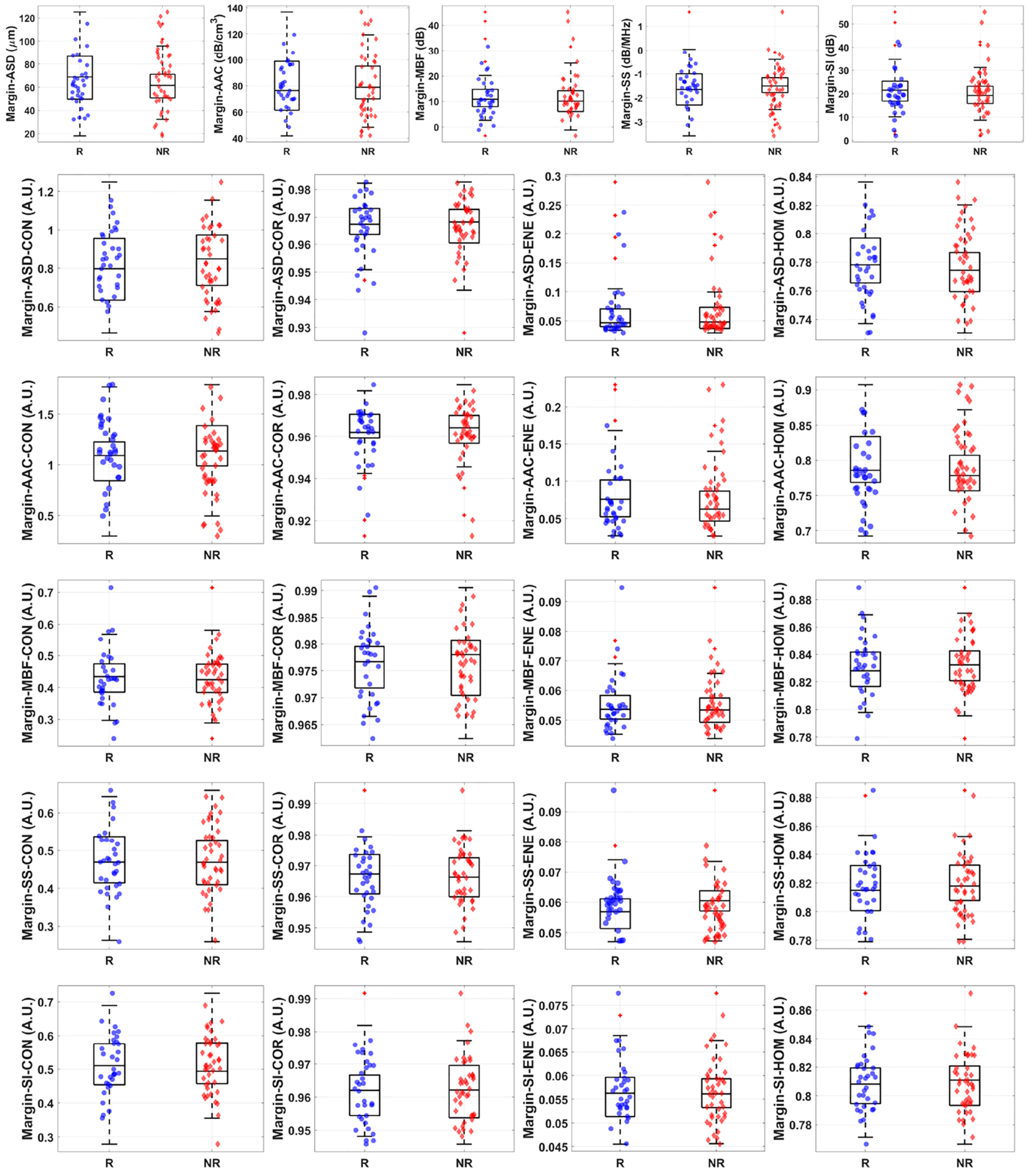

Supplementary Figure 10: Mean-values and texture features from tumour margin.

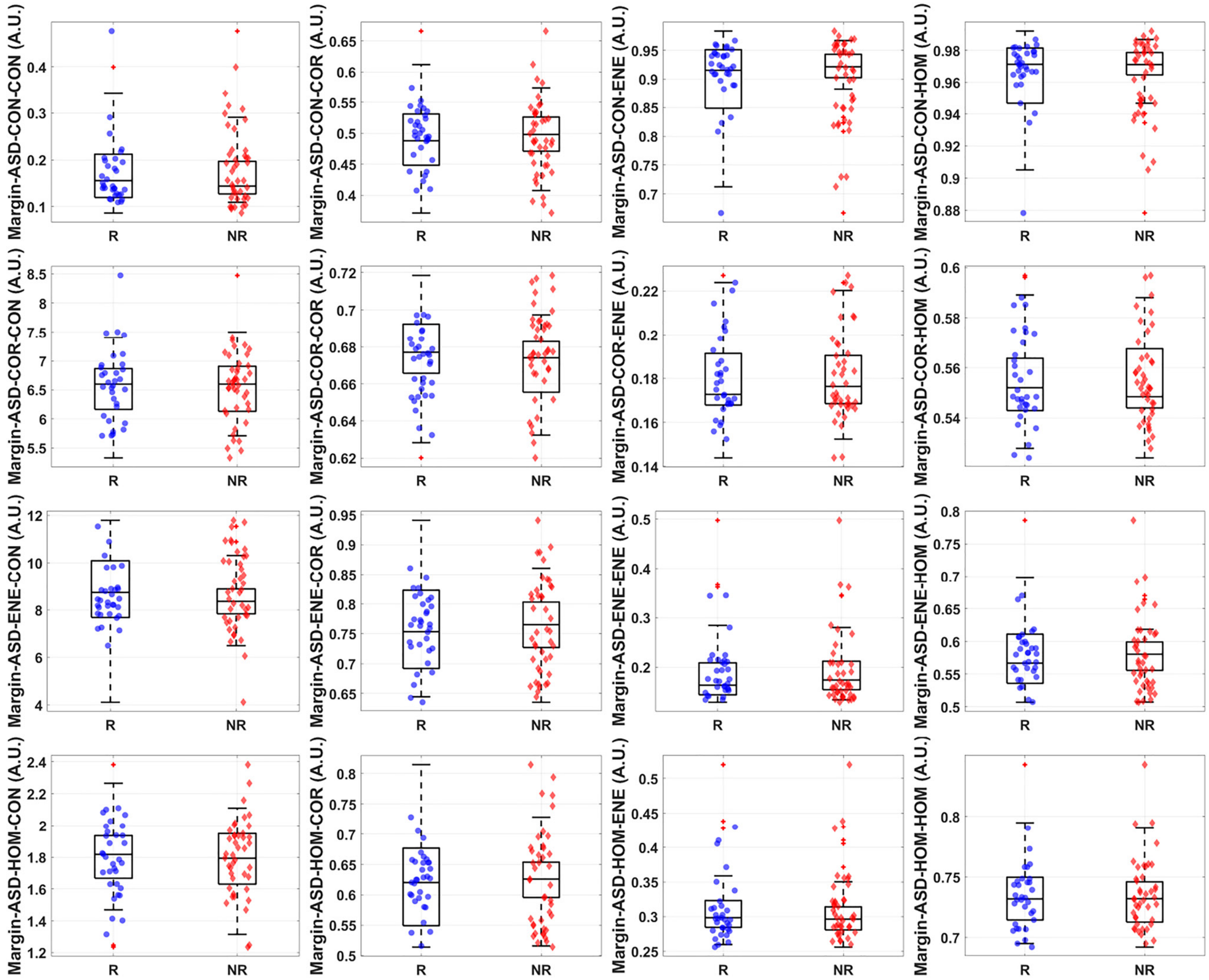

Supplementary Figure 11: ASD texture-derivate features from tumour margin.

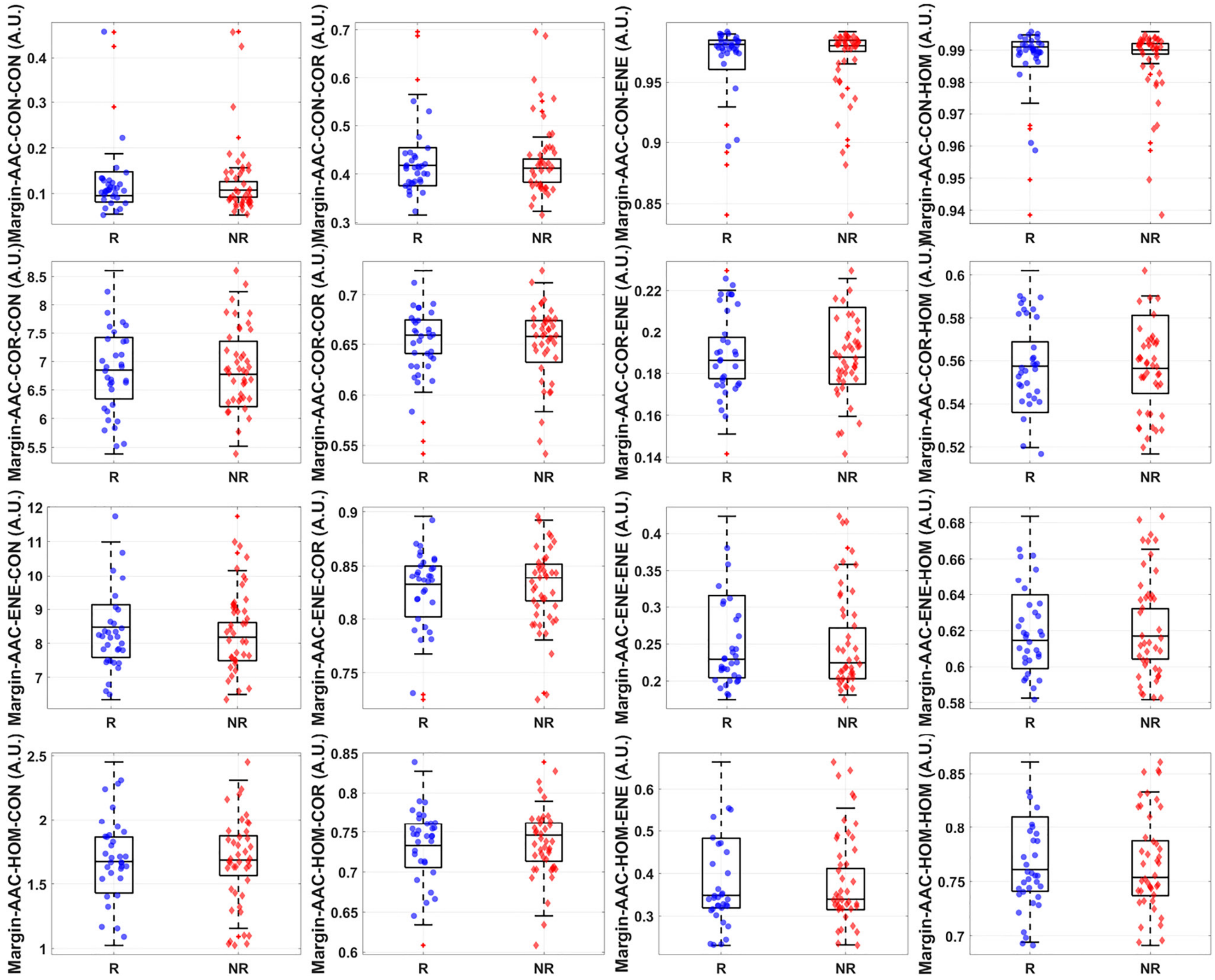

Supplementary Figure 12: AAC texture-derivate features from tumour margin.

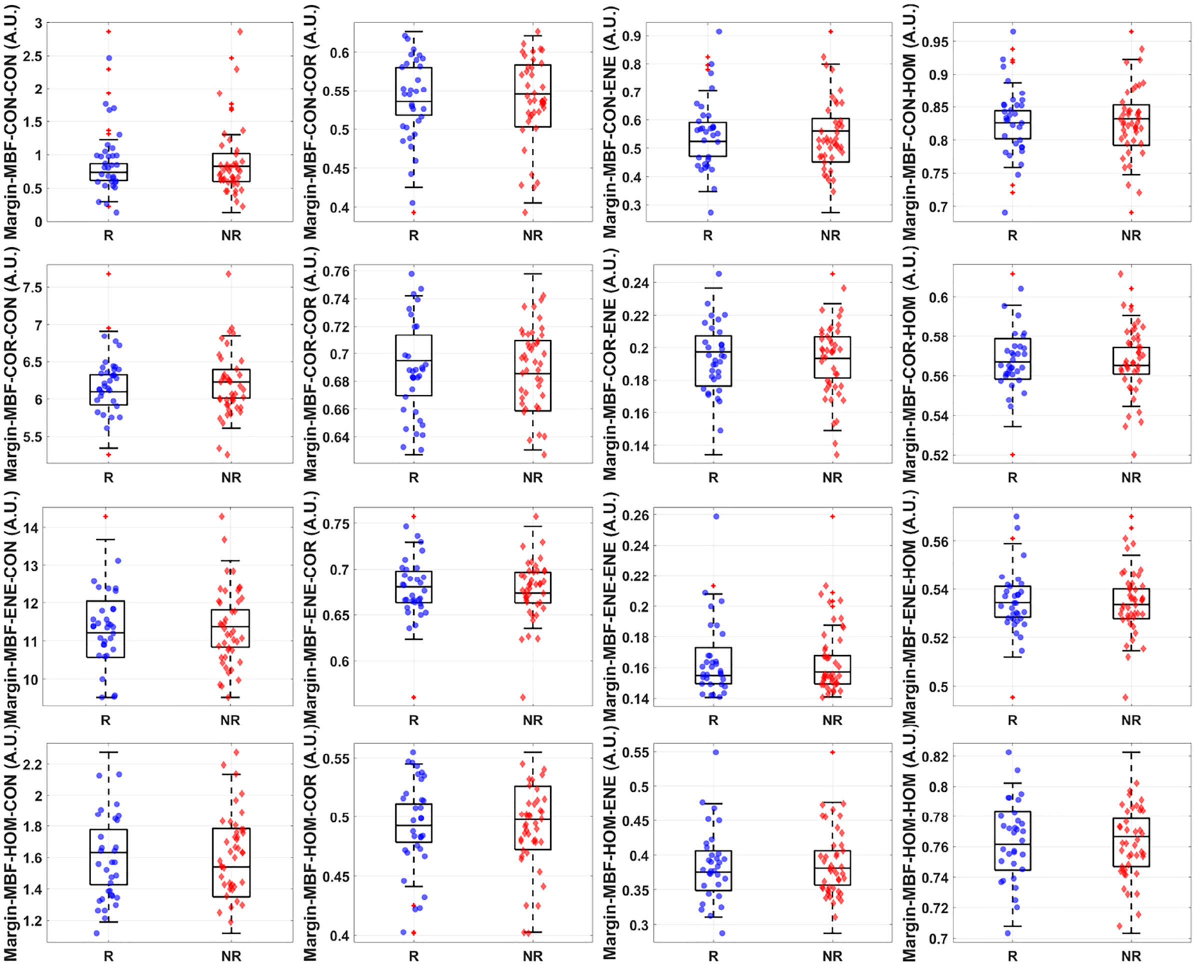

Supplementary Figure 13: MBF texture-derivate features from tumour margin.

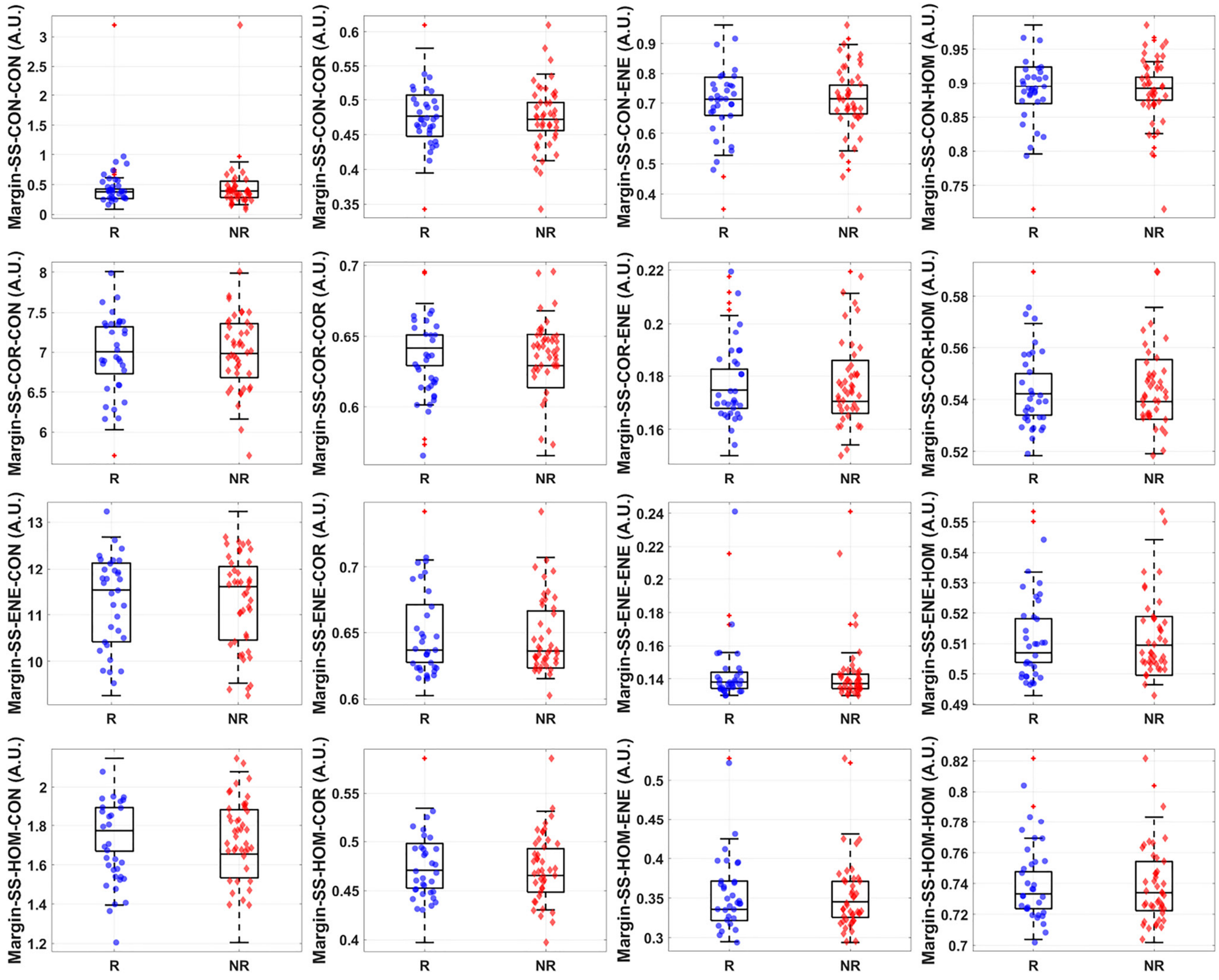

Supplementary Figure 14: SS texture-derivate features from tumour margin.

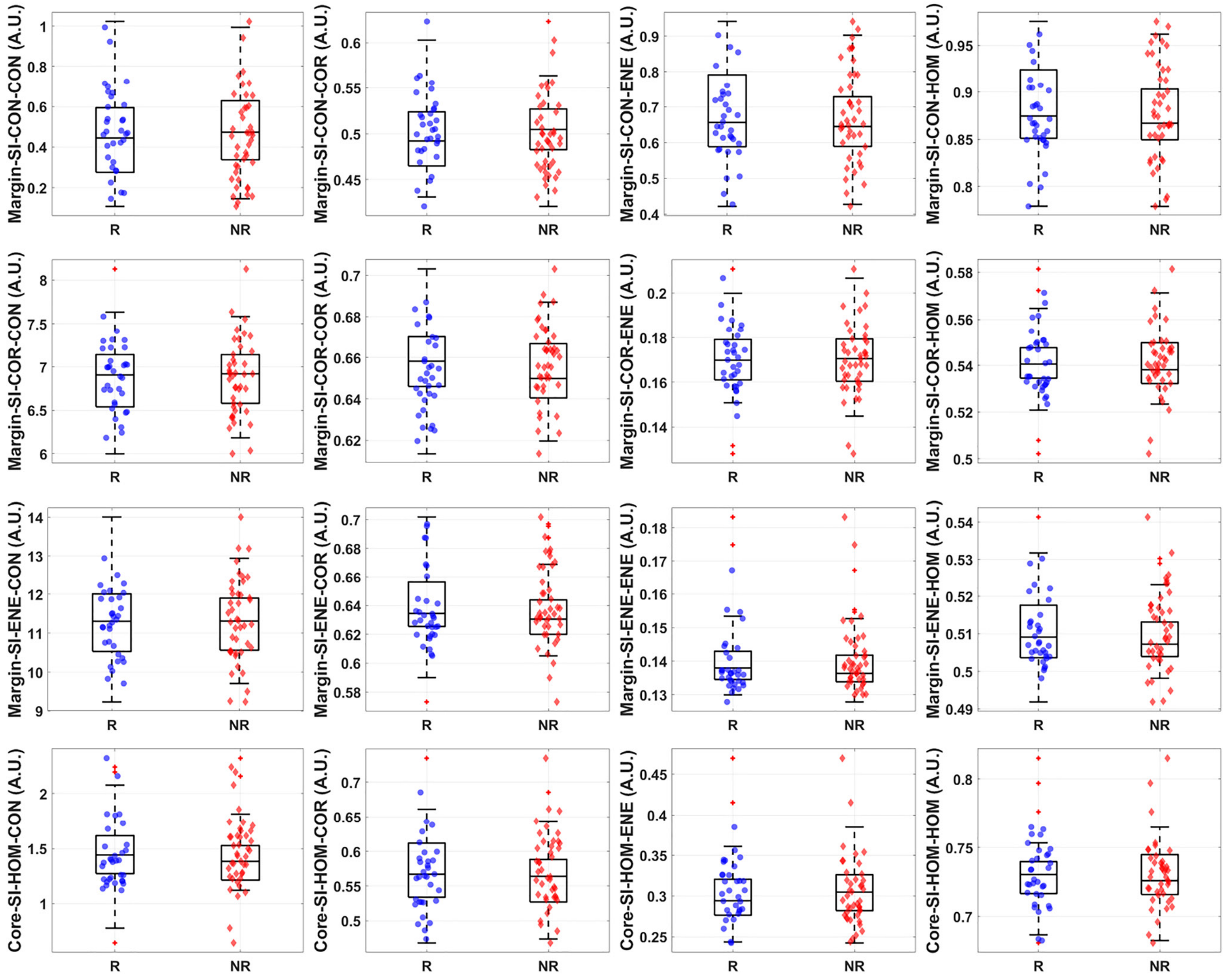

Supplementary Figure 15: SI texture-derivate features from tumour margin.

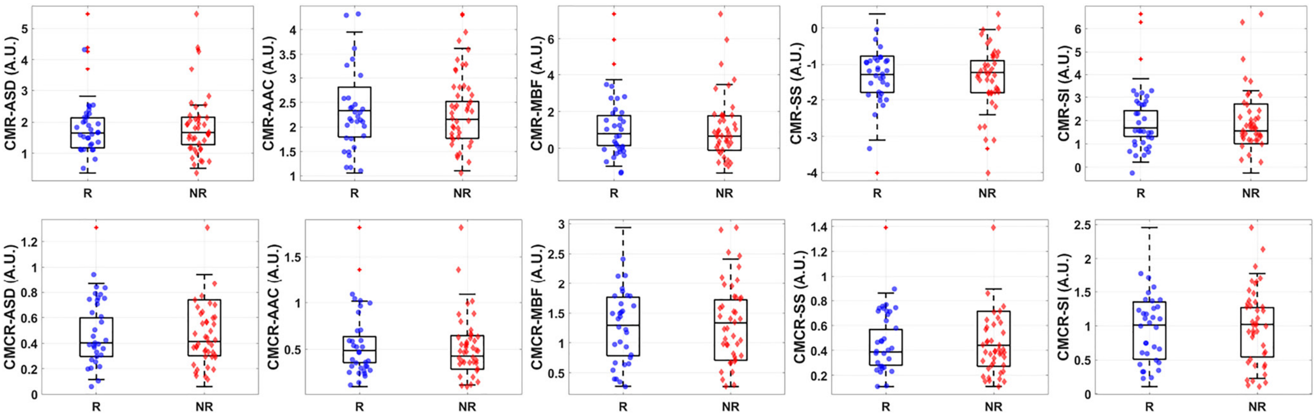

Supplementary Figure 16: CMR and CMCR of QUS parametric images.
